# Determinants of vaccine hesitancy among healthcare workers in an international multicenter study within the EuCARE project

**DOI:** 10.1038/s41598-025-17507-y

**Published:** 2025-08-28

**Authors:** Francis Drobniewski, Marcia Ashmi, Dian Kusuma, Raheelah Ahmad, Daniel Naumovas, Dovilė Juozapaitė, Cristina Toscano, Elvira Perea, Ana B. Abecasis, Miguel Viveiros, Joana P. V. Pereira, Björn-Erik Ole Jensen, Nils Bardeck, Julia Fonseca de Morais Caporali, Jorge Andrade Pinto, Francesca Incardona, Milosz Parczewski, Karol Serwin

**Affiliations:** 1 Department of Infectious Diseases, Faculty of Medicine, Imperial College London, London, W12 0NN UK; 2 Department of Global, Public & Population Health & Policy, School of Health & Medical Sciences, City St George’s University of London, Northampton Square, London, EC1V 0HB UK; 3 Vilnius Santaros Klinikos Biobank, Vilnius University Hospital Santaros Klinikos, Santariskiu st. 2, 08410 Vilnius, Lithuania; 4 VU LSC-EMBL Partnership for Genome Editing Technologies, Life Sciences Center, Vilnius University, Sauletekio al. 7, 10257 Vilnius, Lithuania; 5 Unidade Local de Saúde Lisboa Ocidental-Hospital Egas Moniz, Rua da Junqueira, 126, 1349-019 Lisboa, Portugal; 6 Unidade Local de Saúde Lisboa Ocidental-Hospital de São Francisco Xavier, Estrada do Forte do Alto do Duque, 1449-005 Lisboa, Portugal; 7 Global Health and Tropical Medicine, GHTM, LA-REAL, Instituto de Higiene e Medicina Tropical, IHMT, Universidade NOVA de Lisboa, Lisboa, Portugal; 8 Department of Gastroenterology, Hepatology and Infectious Diseases, Medical Faculty, University Hospital Duesseldorf, Heinrich Heine University, Duesseldorf, Germany; 9 Laboratory for Viral Resistance Research, Institute of Virology, University Hospital of Cologne, Cologne, Germany; 10 School of Medicine and Hospital das Clínicas, Universidade Federal de Minas Gerais, Av. Alfredo Balena, 190, Belo Horizonte, Minas Gerais Brazil; 11 EuResist Network GEIE, Rome, Italy; 12 Department of Infectious, Tropical Diseases and Acquired Immunodeficiency, Pomeranian Medical University, Szczecin, Poland; 13 Present Address: Department of Public Health & Epidemiology, College of Medicine and Health Sciences, Khalifa University of Science and Technology, Abu Dhabi, United Arab Emirates

**Keywords:** Public health, Human behaviour

## Abstract

A total of 2079 healthcare workers (HCWs) responded to an anonymous survey on vaccine hesitancy conducted between December 2022 and October 2023 at centers in Lithuania, Portugal, Poland, Germany, and Brazil. Only 55.7% were confident about the long-term safety of the COVID-19 vaccine; 10% believed the risk of having COVID-19 vaccines was greater than the risk of COVID-19 itself. 54.2% believed that COVID-19 vaccination should be compulsory for all staff working in healthcare settings (unless medically exempt), for all patient-facing HCW (59.0%), and for all medical, nursing, and midwifery students (56.7%); fewer supported compulsory influenza vaccination, i.e., 38.0%, 41.5% and 39.7% for equivalent groups. Under half had been vaccinated for influenza in recent years. Respondents were supportive of childhood vaccinations. Level of education, specific HCW occupation and geography were associated with the degree of vaccination hesitancy. The majority trusted advice from health professionals or scientists/doctors and distrusted statements from politicians. Key factors that HCWs thought would encourage vaccination included: vaccine availability at their workplace, vaccination of professional colleagues, sufficient opportunity to ask about vaccine safety and efficacy, supportive information from international bodies, and HCW plans to visit vulnerable family/friends. Generic vaccine promotion activities, whilst valuable, require nuanced modification for different HCW subgroups, vaccines, education levels, and geography.

**Supplementary Information:**

The online version contains supplementary material available at 10.1038/s41598-025-17507-y.

## Introduction

In 2012, the WHO defined vaccine hesitancy (VH) very broadly as a “complex behavioural phenomenon specific to vaccines, context, time, and place influenced by factors of complacency, convenience and confidence” i.e. a definition of VH which is also pragmatically highly variable depending on the vaccine, context, time and geographical location^1^. Subsequently, in 2019, the WHO described VH as one of the major threats to global health triggered by a significant global increase in a key vaccine-preventable disease, measles^2^. Vaccine hesitancy has contributed to measles cases and outbreaks, not only, as might be expected, in lower-middle-income countries (LMICs) but in high-income countries including the USA, Europe and the UK^3–6^.

Increase in the incidence, and frequency of outbreaks of measles and other vaccine-preventable diseases reflects an increasing failure to achieve herd or collective immunity in populations and VH has been identified as the key reason for this^7,8^.

Since COVID-19, while WHO appropriately listed access to vaccines as a key health issue to track toward recovery in 2021, the COVID-19 pandemic emphasized the problem of VH. Novel vaccines were being developed rapidly to be used initially in older adults rather than children against a disease with low morbidity and mortality in children and young adults. A recent Cochrane Review^9^ confirmed that compared to placebo, most vaccines reduce, or likely reduce, the proportion of participants with confirmed symptomatic COVID-19, and for some, there is good evidence that they reduce severe or critical disease. Equally, there was little or no difference between most vaccines and placebo for serious adverse events^9^. Even amongst clinical and scientific experts there can be significant VH. For example, COVID-19 VH was found in UK HCWs and in postgraduate science, medical and nursing students in a UK-based study; Asian and Black students were 2 and 3.2 times less likely to accept the COVID-19 vaccine than White students^10^. Even among healthcare workers (HCW) in high income countries, there remained a lack of consensus, which required policy makers to reconsider the balance between autonomy, occupational health and public health, given the sub-optimal uptake of COVID-19 vaccines^11,12^. In a review of 39 studies of VH in HCWs published from 2015 to 27 May 2021, only 5 studies quantified VH, which varied from 3% (USA) to 17% (Croatia) and to 11% and 44% (both in France)^11^. VH in HCW is an important metric to track and understand as it has major health, social, cultural and economic implications with the potential to increase public doubt and jeopardise healthcare provision.

One potential difficulty in comparing studies is that VH can be conceptualized in different ways^8^. One of the main-stream approaches to conceptualize VH is using the “Three Cs”: confidence, complacency and convenience.

“Confidence” is defined as the trust that people have in vaccine, the healthcare system itself, and the process leading to decisions on licensing. Bussink-Voorend^8^ commented that only a “few studies describe the (lack of) trust or confidence as a component of VH^13–15^.

“Complacency” is the individual evaluation of the risks and benefits of vaccines where in relation to VH there is a tendency to over-estimate vaccination risks whilst under-estimating the risks of the vaccine-preventable disease^13,16^. “Convenience” concerns availability, accessibility of vaccines, user-friendliness and delivery of vaccines.

This international multicenter study analysed the degree of VH, in HCWs, with a focus on COVID-19 and influenza vaccination, including acceptance of mandatory vaccination for HCWs. Through theoretical grounding using the Three Cs approach, we aimed to identify factors associated with vaccine uptake (and vaccine hesitancy) which can inform development of policies and interventions to increase vaccine confidence, and which are generalisable, to improve pandemic and wider public health planning.

## Materials and methods

The study was carried out within the framework of the “European Cohorts of Patients and Schools to Advance Response to Epidemics-EuCARE” project (www.eucareresearch.eu).

We (FD, MA, RA, DK) developed a modified totally anonymous questionnaire building on an initial questionnaire on vaccine hesitancy developed and validated previously^10^ and the Three Cs described above. Within the questionnaire we incorporated, with minor modifications, the key Likert-type questions relating to vaccines of the Vaccine Hesitancy Scale described by Larson^17^ and subsequently validated by Shapiro^18^. A key objective was to identify using the theoretical grounding encapsulated in the Three Cs approach to identify factors associated with vaccine uptake amongst HCWs.

The following EuCARE partners participated: Vilnius University Hospital Santaros Klinikos, Lithuania; CHLO, Lisboa, and Instituto de Higiene e Medicina Tropical, IHMT, Universidade NOVA de Lisboa, Lisboa, Portugal; Medical Faculty and University Hospital Duesseldorf, Heinrich Heine University, Duesseldorf, Germany; School of Medicine and Hospital das Clínicas, Federal University of Minas Gerais Brazil; Pomorski Uniwersytet Medyczny w Szczecinie, Poland. The questionnaire was distributed to over 25,000 HCWs. Center-specific numbers were: Brazil (2500), Germany (8000), Lithuania (6060), Portugal (6534) and Poland (2000). The participants were hospital-based HCWs affiliated with a given university hospital.

Questionnaires were translated into local languages (Polish, Lithuanian, Portuguese, Brazilian-Portuguese, German) by a lead member from each participating center which was reviewed by a second within-country team member for accuracy and clarity. Staff titles/grades were modified if necessary to ensure that the same “job-work profile” was brought together e.g. doctors, midwives (e.g. in the UK where they may be nurses first and then become midwives rather than starting as career midwives).

The Imperial College London research ethics committee approved the study as well as City University Research Ethics Committee (Ref: ETH2021-0904). Imperial supported EuCARE partners in Lithuania, Germany, Portugal, Poland and Brazil in obtaining ethics permission or waiver: the revised and finalized questionnaire, protocol, participant investigation sheet in English and relevant local language were approved in their ethical review processes to conduct a multicenter prospective study. Informed consent was obtained from all participants. All methods were performed in accordance with the relevant guidelines and regulations.

The survey was conducted using a self-administered, anonymous questionnaire sent out to all healthcare workers of the participating organisations through their respective Human Resources and IT departments by email and QR code. HR departments sent out the questionnaire using all-staff mailing within their organization. The questionnaire could be answered in any of the above languages if desired, in addition to the center-appropriate one. The questionnaire covered basic demographics (as permitted by ethics committees), knowledge of COVID-19 and influenza vaccines, other vaccines and their willingness to receive these vaccines. Participants answered questions regarding their view on safety, side-effects, efficacy of COVID-19, influenza and vaccines in general. We also questioned whether they believed that healthcare workers (including students) should receive COVID-19 or influenza vaccinations compulsorily. More importantly, we inquired about the factors that would motivate them to get vaccinated (and those that made them less likely to do so) using questions based on the Three Cs theoretical approach. Wherever possible questions were framed so that meaningful answers could be obtained regardless of whether an individual had been vaccinated e.g. “what would encourage/help you to be vaccinated?” and “what did help/encourage you to be vaccinated?”. We employed a six-point Likert scale to obtain a more nuanced response for some questions. Free text options were also offered.

Participants received the link by email (or QR code) from which they could enter data into the survey anonymously. The study was conducted from December 2022 to October 2023 i.e. within the COVID-19 pandemic period but specifically after effective COVID-19 vaccines had been developed. Two reminders were sent out.

### Analysis

We conducted descriptive and multivariate regression analysis. For descriptive analyses, we provided the characteristics and prevalence of participants who responded affirmatively (agree/strongly agree or yes). For regression analyses, we used multivariate logistic regression. We explored whether education level, healthcare worker (HCW) occupation, or country of origin impacted the importance of the above factors and whether there were national differences. All analyses were conducted using Excel and STATA MP 15.1.

## Results

### Participants

Overall, 2079 HCWs responded with complete demographic information for regression analysis relating to job, education and place of work for 1907 (Table 1). Only a minimal common dataset was permitted across all the study sites by all the ethics committees. Over a third of respondents (34.9%) were medical doctors.

The distribution of respondents by country was: Lithuania—332, Portugal—409, Poland—445, Brazil—247, and Germany—583. An additional 63 responses were not linked to a specific center. The corresponding response rates were 5.5% for Lithuania, 6.3% for Portugal, 22.3% for Poland, 9.9% for Brazil, and 7.3% for Germany. A core data set for subgroup analysis (*n* = 1907) was used where individuals had given complete information on their occupation, education level and country of work.

**Table 1:** Descriptive statistics of core multinational HCW cohort.

	*n*	%
(a) Characteristics		
Education level		
Doctorate	464	24.3
Master	685	35.9
Bachelor	347	18.2
High school	411	21.6
Occupations		
Doctors	669	34.92
Allied health professionals	545	28.44
Administrations	221	11.53
Scientists	129	6.73
Academics	100	5.22
Midwives	97	5.06
Nurses	87	4.54
Others	68	3.55
Country of work		
Brazil	237	12.4
Germany	537	28.2
Lithuania	298	15.6
Poland	408	21.4
Portugal	360	18.9
Others	67	3.5

There were *n* = 1907 HCW with all variables.

“Other countries” could not be attributed to the main participating centers; individuals came from Italy, Afghanistan, UK, Mozambique, etc.

Overlap of roles was minimised by staff at each center translating staff/job roles and positions. Clinical and laboratory scientists who defined their work as clinic or hospital based were categorized as “scientists,” while “academics” referred to individuals primarily involved in university-based research or teaching.

### COVID-19 vaccination

The majority, 96.8% (*n* = 2006) of participants had received COVID-19 vaccination (leaving just 3.2% (*n* = 67) unvaccinated). Messenger RNA-based vaccines were the dominant vaccine types: in our study, 56.5% of respondents received the Pfizer-BioNTech vaccine, and 17.8% received the Moderna vaccine; 15.3% of respondents received the AstraZeneca vaccine, with smaller percentages for other vaccines. The majority of respondents had received 2, 3 or 4 doses/boosters (23.8% 36.2% and 26.8%, respectively).

While the COVID-19 vaccination rates were high, just over two thirds (67.90% *n* = 1301) felt that the “COVID-19 vaccination available” to them was safe; 14.7% (*n* = 282) did not feel the offered vaccines were safe; 14.0% (*n* = 268) were “not sure” and 3.4% (*n* = 65) that only some available were safe (Pfizer-BioNTech, Moderna, Coronovac, Spikevac, Johnson and Johnson). Considering further questions around COVID-19 vaccine safety, 45.2% (*n* = 866) had concerns as to whether they had been adequately tested and 9.6% (*n* = 183) preferred to obtain their own immunity from getting COVID-19 itself. Specifically, only 55.7% (*n* = 1068) were “confident about the long-term safety of the COVID-19 vaccine offered” to them with 19.1% (*n* = 365) indicating lack of confidence, and a similar proportion, 23.4% (*n* = 448) uncertain with the long-term safety. Individuals were also concerned (27.2%, *n* = 522) about the immediate/short-term side effects of the COVID-19 vaccine, 10% of those responding (*n* = 191) believed that the risk of having COVID-19 vaccines was greater than the risk of COVID-19 itself.

When the vaccinated were asked whether they “believed that the COVID-19 vaccines are effective (work well)” 12.7% (n = 243) answered “no” and 17.8% (n = 340) were not sure. The reasons given by the small number of individuals (n = 67) who were not COVID-19 vaccinated mirrored this to some extent: respondents were able to offer multiple reasons for non-vaccination but 14.6% felt it was “a novel vaccine and has been rushed through the development phases” and 11.1% said they “don’t believe it is helpful in preventing COVID-19”. Other major reasons given were “I did not want it” (16.1%); “not offered” (9.1%); “fear of side-effects” (12.6%); “not at great risk from COVID-19” (6.0%); “preferred to take the risk of catching COVID-19” (4.5%); “believe I may be allergic to a component of the vaccine” (4.0%).

### Correlates of vaccine hesitancy

Level of education was associated with the degree of vaccination hesitancy. HCWs with Master’s degrees had similar levels of concern regarding the safety and testing of COVID-19 vaccination to those with doctorate-level degrees. However, there was a significantly increased concern in HCWs where the highest qualification was a bachelor’s degree and those with a high school education only (Table 2).

Specific HCWs occupation also had an impact. When compared to medical doctors, allied health professionals and scientists had similar levels of concern but concern (e.g. worry about safety and testing) was significantly higher amongst nurses and administrative staff (Table 2).

Broadly similar rates of concern regarding VH were seen in HCWs in Lithuania, (which received the questionnaire first), Germany and Poland, with lower rates seen in Portugal and Brazil (Table 2).

**Table 2:** Correlates of COVID-19 vaccine hesitation.

	Worry on safety	Worry not adequately tested	Prefer own immunity from getting COVID-19
Variables	AOR (SE)	AOR (SE)	AOR (SE)
Education level			
Doctorate	Ref		
Masters	1.10	1.08	0.98
	(0.16)	(0.14)	(0.24)
Bachelor	2.18***	1.94***	2.37***
	(0.40)	(0.33)	(0.68)
High school	2.39***	2.44***	2.26***
	(0.40)	(0.39)	(0.58)
Occupations			
Doctors	Reference		
Allied health	1.11	1.25	1.32
	(0.15)	(0.16)	(0.28)
Administration	1.76***	1.73***	1.57
	(0.33)	(0.30)	(0.45)
Scientists	1.33	1.18	1.16
	(0.30)	(0.24)	(0.46)
Academics	1.51	1.45	2.23**
	(0.39)	(0.34)	(0.81)
Midwives	0.96	0.92	0.91
	(0.26)	(0.23)	(0.42)
Nurses	1.95***	1.86**	1.51
	(0.50)	(0.47)	(0.58)
Others	1.34	1.23	1.08
	(0.39)	(0.33)	(0.50)
Country			
Lithuania	Reference		
Brazil	0.19***	0.36***	0.26***
	(0.05)	(0.07)	(0.12)
Germany	0.98	0.85	1.57
	(0.17)	(0.14)	(0.45)
Poland	0.96	1.01	1.62
	(0.16)	(0.16)	(0.46)
Portugal	0.32***	0.65**	0.70
	(0.06)	(0.11)	(0.22)
Others	0.63	0.71	2.14
	(0.19)	(0.20)	(0.91)
Observations	1,907	1,907	1,907

AOR, adjusted odds ratio, SE, Standard errors, ***p < 0.01, **p < 0.05.

### Sources of vaccine information

**Fig. 1:**
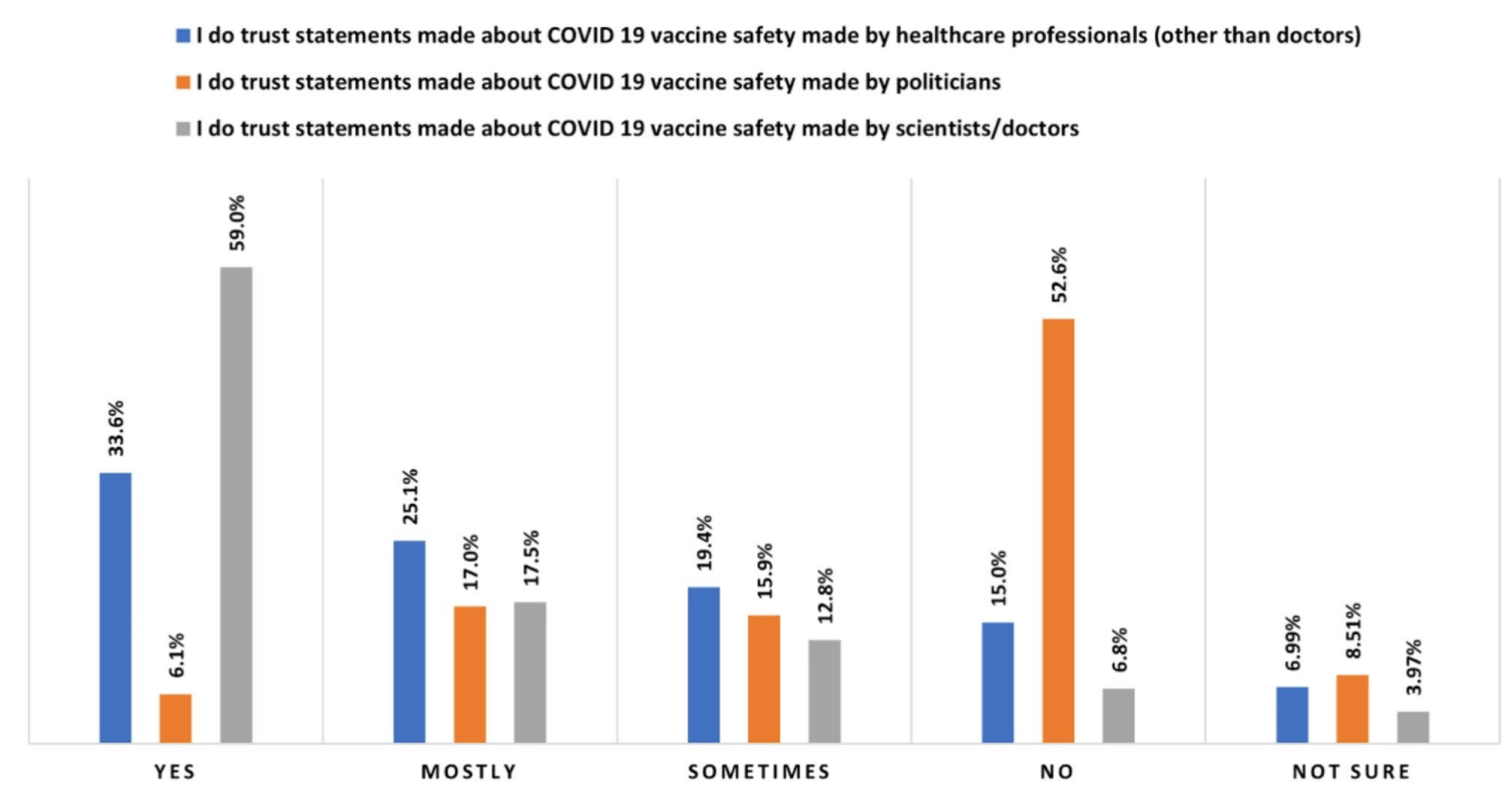
Respondents trust in COVID-19 vaccine safety information from different sources.

In terms of information sources about COVID-19 vaccination that were most or least trusted (Fig. 1) the majority agreed with the statements “I do trust statements made about COVID-19 vaccine safety made by health professionals or scientists/doctors” (76.5% “trusting” and “mostly” trusting). There was an equally strong negative response to trusting statements “made by politicians” (23.1% “trusting” or “mostly trusting; 52.6% “not” trusting at all). Similar proportions were seen in relation to efficacy (data not shown).

### Other vaccines

Overall respondents were very supportive of the value of childhood vaccinations and their safety in particular; 80.0% (*n* = 1500) believed them to be safe with only 5.2% (*n* = 98) not believing they were safe and 12.3% (*n* = 231) not sure. An additional 2.5% (*n* = 47) felt that some childhood vaccines were unnecessary.

We enquired as to whether individuals would like to have an influenza vaccination this year and their influenza vaccination history in previous years. Of those responding only 13.7% (*n* = 113) said yes, with 67.0% (*n* = 555) saying no and 14.0% not sure and 5.3% of those asked (*n* = 44) had already had an influenza vaccination at the time of asking. Only about half of HCWs had had influenza vaccination in previous years (Table 3).

**Table 3:** Influenza vaccination history of survey respondents.

	Yes (%) *n*	No (%) *n*	Cannot remember (%) *n*	Not available in country (%) *n*
In autumn/winter of 2021	54.0% 1027	43.6% 829	2.4% 45	0.1% 2
In autumn/winter of 2020	52.8% 1005	43.4% 826	3.6% 68	0.2% 4
Any year BEFORE COVID-19 (i.e. 2019 or before)	52.0% 990	43.1% 820	4.7% 89	0.2% 4

**Fig. 2:**
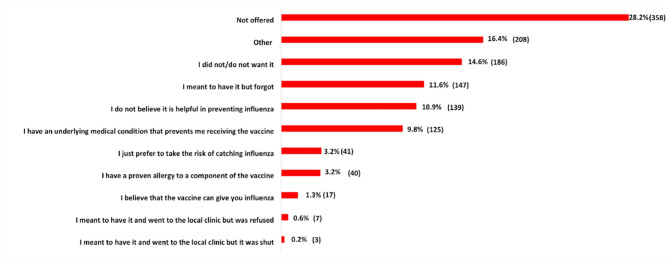
Reasons for not taking the influenza vaccine.

The range of reasons provided by those not taking the influenza vaccine in the past or not planning to accept vaccination this year are presented in Fig. 2; the most common reason being the vaccine was not specifically offered to them (i.e. only a general offer that influenza vaccine was available) and 10.9% did not believe the vaccine to be effective in preventing influenza. A small proportion (1.3%) believed that the vaccine would cause influenza.

#### Compulsory vaccination

Views on compulsory COVID-19 and influenza vaccination show less consensus for influenza than COVID-19 (Table 4).

**Table 4:** Attitude of HCWs to compulsory COVID-19 and influenza vaccination.

“It should be compulsory…”	Yes % (*n*)	No % (*n*)	Not sure % (*n*)
for all medical, nursing and midwifery students to be vaccinated against COVID-19 (unless medically exempt)?	56.7% (1131)	28.3% (564)	15.0% (300)
for all patient-facing healthcare staff to be vaccinated against COVID-19 (unless medically exempt)?	59.0% (1177)	28.1% (560)	13.0% (258)
for all staff working in healthcare settings to be vaccinated against COVID-19 (unless medically exempt)?	54.2% (1082)	32.2% (643)	13.5% (270)
for all medical, nursing and midwifery students to be vaccinated against influenza (unless medically exempt)?	39.7% (745)	43.1% (810)	17.1% (321)
for all patient-facing healthcare staff to be vaccinated against influenza (unless medically exempt)?	41.5% (779)	42.6% (800)	15.8% (297)
for all staff working in healthcare settings to be vaccinated against influenza (unless medically exempt)?	38.0% (714)	46.6% (874)	15.4% (288)

Over half of respondents felt that COVID-19 vaccination should be compulsory for all staff working in healthcare settings (unless medically exempt) (54.2%), for all patient-facing healthcare staff (59.0%) and for all medical, nursing and midwifery students (56.7%) (Table 4).

Far fewer supported compulsory influenza vaccination i.e. compulsory for all staff working in healthcare settings (38.0%, for all patient-facing healthcare staff (41.5% and for all medical, nursing and midwifery students (39.7%) (Table 4).

Table 5 examines compulsory vaccination more closely in relation to reference groups (there was complete data for education, occupation, and country for comparison for 1907 participants). There was widespread equanimity for compulsory vaccination of HCWs for COVID-19 and influenza.

We examined whether any HCW subgroups differed significantly in their views on this. Individuals with a high school education or a bachelor’s degree were less likely to support mandatory COVID-19 or influenza vaccination compared to those with a doctorate degree. By professional group, nurses were less supportive of compulsory vaccination.

There was widespread support for mandatory childhood vaccination but administrative staff and academics had a higher probability of concern with the safety of childhood vaccinations.

Compared to Lithuania as a reference country on compulsory vaccination, Brazil was more likely to support compulsion for both COVID-19 and influenza vaccination whilst Germany leant the other way.

**Table 5:** Correlates of disbelief on mandatory vaccines against COVID-19 and Influenza.

Variables	Disbelief 1	Disbelief 2	Disbelief 3	Disbelief 4	Disbelief 5	Disbelief 6	Disbelief 7 Child vax
	AOR (SE)	AOR (SE)	AOR (SE)	AOR (SE)	AOR (SE)	AOR (SE)	AOR (SE)
Education level							
Doctorate	Reference						
Masters	0.88	0.92	0.89	0.97	0.89	0.95	0.75
	(0.13)	(0.14)	(0.13)	(0.13)	(0.12)	(0.13)	(0.24)
Bachelor	1.34	1.47**	1.51**	1.46**	1.55**	1.42**	2.39**
	(0.25)	(0.27)	(0.27)	(0.25)	(0.27)	(0.25)	(0.85)
High school	1.45**	1.55***	1.39**	1.58***	1.55***	1.32	1.35
	(0.24)	(0.26)	(0.23)	(0.25)	(0.25)	(0.21)	(0.46)
Qualification							
Doctors	Reference						
Allied health	1.05	1.14	1.18	1.13	1.11	1.17	1.16
	(0.14)	(0.16)	(0.16)	(0.15)	(0.14)	(0.15)	(0.34)
Administration	1.29	1.34	1.36	1.20	1.23	1.25	2.53**
	(0.24)	(0.25)	(0.25)	(0.21)	(0.22)	(0.22)	(0.92)
Scientists	1.06	1.17	1.24	1.45	1.32	1.38	1.52
	(0.24)	(0.27)	(0.28)	(0.30)	(0.27)	(0.29)	(0.73)
Academics	1.15	1.10	1.09	1.31	0.98	1.07	3.15**
	(0.30)	(0.30)	(0.29)	(0.32)	(0.25)	(0.27)	(1.41)
Midwives	0.73	0.84	1.04	1.15	1.06	1.09	0.79
	(0.20)	(0.23)	(0.27)	(0.28)	(0.26)	(0.27)	(0.51)
Nurses	1.75**	1.93**	1.63	1.53	1.63	1.42	1.47
	(0.45)	(0.50)	(0.42)	(0.39)	(0.41)	(0.36)	(0.69)
Others	0.58	0.68	0.84	1.01	0.95	1.00	1.06
	(0.20)	(0.23)	(0.26)	(0.28)	(0.27)	(0.28)	(0.68)
Country							
Lithuania	Reference						
Brazil	0.39***	0.36***	0.32***	0.29***	0.27***	0.24***	0.06***
	(0.09)	(0.08)	(0.07)	(0.06)	(0.06)	(0.05)	(0.05)
Germany	1.44**	1.38	1.87***	1.50**	1.58***	2.29***	0.97
	(0.26)	(0.25)	(0.33)	(0.25)	(0.27)	(0.39)	(0.33)
Poland	0.72	0.75	0.76	0.86	0.91	0.85	1.06
	(0.13)	(0.14)	(0.14)	(0.14)	(0.15)	(0.14)	(0.34)
Portugal	0.89	0.75	0.78	1.38	1.24	1.23	0.14***
	(0.17)	(0.14)	(0.14)	(0.23)	(0.21)	(0.21)	(0.07)
Others	0.84	0.76	0.80	0.97	0.92	0.74	1.00
	(0.27)	(0.25)	(0.25)	(0.29)	(0.28)	(0.23)	(0.56)
Observations	1907	1907	1907	1867	1867	1867	1821

Disbelief 1 = Not believe it should be compulsory for all medical, nursing, and midwifery students to be vaccinated against COVID-19.

Disbelief 2 = Not believe it should be compulsory for all patient-facing healthcare staff to be vaccinated against COVID-19.

Disbelief 3 = Not believe it should be compulsory for all staff working in healthcare settings to be vaccinated against COVID-19.

Disbelief 4 = Not believe it should be compulsory for all medical, nursing and midwifery students to be vaccinated against influenza.

Disbelief 5 = Not believe it should be compulsory for all patient-facing healthcare staff to be vaccinated against influenza.

Disbelief 6 = Not believe it should be compulsory for all staff working in healthcare settings to be vaccinated against influenza.

Disbelief 7 = Not believe in the safety of childhood vaccinations.

AOR, Adjusted odds ratio, SE, Standard errors, ***p < 0.01, **p < 0.05.

**Table 6:**
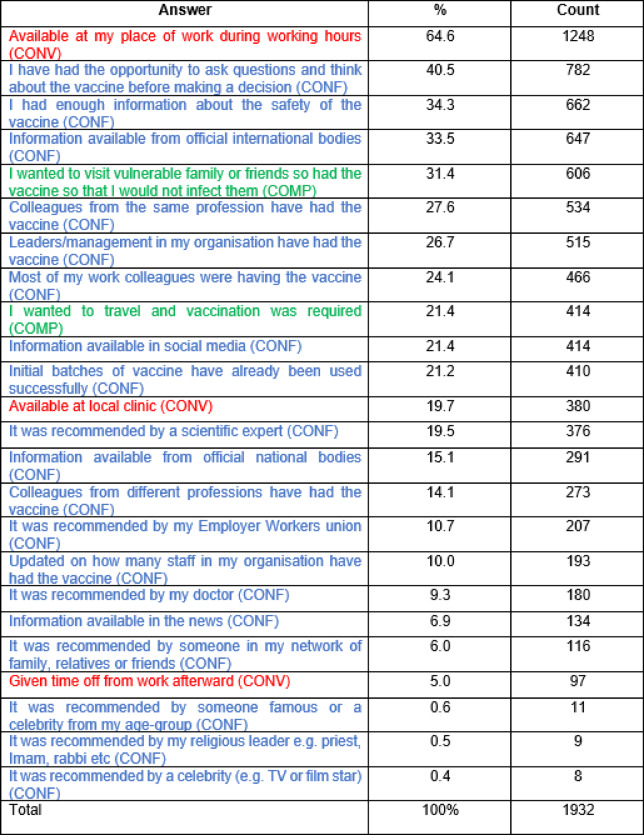
What helped persuade respondents to be vaccinated against COVID-19-structured and colour coded to the three Cs.

 Table 6 is colour coded based on the “Three Cs” i.e. Red for Convenience (practical, access issues) (CONV); Blue for Confidence (CONF) and Green for Complacency (COMP).

Within the HCW cohort, we explored whether education level, individual healthcare worker occupation, or country of origin had an impact on the importance of the above factors and whether there were national differences (Table 7). Regarding education, compared to participants with doctoral-level education, those with lower education levels, including high school and bachelor’s degrees, had significantly higher odds of reporting that “Opportunity to ask questions and think about the vaccine before making a decision” and “Information available from official international bodies” were not factors in getting COVID-19 vaccines (AOR > 1). Those holding a bachelor’s degree had significantly higher odds of reporting that “enough information about the safety of the vaccine” was not associated with COVID-19 vaccination whilst “wanting to visit vulnerable family or friends” was more likely to be associated.

Other factors appeared to be similarly reported across education levels. By professional qualifications, nurses and allied health workers showed significantly higher odds of reporting that “Enough information about the safety of the vaccine” and “Information available from official international bodies” were not factors in getting COVID-19 vaccines, compared to doctors. Additionally, scientists and midwives had higher odds of reporting that “Available at my place of work during working hours” was not a factor in getting COVID-19 vaccines, compared to doctors.

Key influencing strategies showed significant geographical disparities. For example, compared to Lithuania, HCWs in Poland, Portugal, and other countries, showing significantly higher odds of reporting that vaccine availability at their place of work was not a factor in getting COVID-19 vaccines. In contrast, individuals in Brazil, Portugal and Germany showed higher odds of reporting that “Opportunity to ask questions and think about the vaccine before making a decision” and “Information available from official international bodies” were significant factors in getting COVID-19 vaccines (AOR < 1).

HCWs in Germany and Portugal, reported that “leaders/management in their organisation having a vaccine” were significant factors in promoting vaccination.

**Table 7:** Characteristics associated with reporting selected motivators as ‘not a factor’ in COVID-19 vaccination decisions.

Variable	Available at workplace	Opportunity to ask questions	Enough info on safety	Info from international bodies	Visiting vulnerable relatives	Colleagues vaccinated	Leaders vaccinated	Most work colleagues vaccinated
	AOR (SE)	AOR (SE)	AOR (SE)	AOR (SE)	AOR (SE)	AOR (SE)	AOR (SE)	AOR (SE)
Education level								
Doctorate	Reference							
Masters	0.98	1.18	0.91	1.13	0.80	0.78	0.71**	0.82
	(0.13)	(0.16)	(0.12)	(0.15)	(0.11)	(0.11)	(0.11)	(0.12)
Bachelor	1.15	1.55**	1.59**	1.73***	0.67**	1.08	0.87	0.79
	(0.20)	(0.27)	(0.29)	(0.30)	(0.12)	(0.21)	(0.17)	(0.15)
High school	1.27	1.87***	1.36*	1.81***	0.87	0.81	0.72	0.93
	(0.21)	(0.30)	(0.23)	(0.30)	(0.15)	(0.15)	(0.13)	(0.17)
Qualification								
Doctors	Reference							
Allied health	0.96	0.97	1.39**	1.31**	1.01	1.04	0.95	1.04
	(0.13)	(0.13)	(0.19)	(0.18)	(0.14)	(0.15)	(0.13)	(0.15)
Administration	1.24	1.07	1.40	1.27	1.22	1.00	0.73	1.42
	(0.21)	(0.19)	(0.26)	(0.23)	(0.23)	(0.19)	(0.14)	(0.30)
Scientists	1.71***	0.79	1.02	0.85	0.59**	1.12	1.24	1.15
	(0.35)	(0.17)	(0.21)	(0.18)	(0.12)	(0.26)	(0.31)	(0.29)
Academics	0.99	0.99	0.83	1.32	1.02	0.68	0.66	0.78
	(0.24)	(0.24)	(0.20)	(0.32)	(0.26)	(0.16)	(0.17)	(0.20)
Midwives	1.79**	0.71	0.98	0.97	0.92	0.99	0.74	1.03
	(0.42)	(0.17)	(0.23)	(0.23)	(0.23)	(0.28)	(0.19)	(0.28)
Nurses	1.33	1.19	1.73**	3.02***	1.73	0.92	0.90	0.94
	(0.33)	(0.35)	(0.47)	(1.08)	(0.50)	(0.25)	(0.27)	(0.25)
Others	2.21***	1.16	1.69	1.48	1.07	1.77	1.16	1.29
	(0.59)	(0.34)	(0.52)	(0.45)	(0.31)	(0.60)	(0.38)	(0.43)
Country								
Lithuania	Reference							
Brazil	0.92	0.14***	1.93***	0.49***	1.99***	1.02	1.02	1.81**
	(0.18)	(0.03)	(0.39)	(0.10)	(0.43)	(0.21)	(0.25)	(0.42)
Germany	1.06	0.25***	1.16	0.65**	0.65**	1.65***	0.37***	0.92
	(0.19)	(0.05)	(0.20)	(0.12)	(0.11)	(0.32)	(0.08)	(0.17)
Poland	2.38***	0.77	0.85	1.78***	0.93	0.53***	0.79	0.71
	(0.40)	(0.15)	(0.14)	(0.33)	(0.16)	(0.09)	(0.16)	(0.13)
Portugal	1.42**	0.25***	2.52***	0.59***	1.60**	1.19	0.42***	2.03***
	(0.25)	(0.05)	(0.47)	(0.11)	(0.30)	(0.23)	(0.08)	(0.43)
Others	2.40***	0.78	1.78	1.30	1.23	1.29	0.90	1.42
	(0.69)	(0.25)	(0.54)	(0.42)	(0.38)	(0.42)	(0.32)	(0.49)
Observations	1,907	1,907	1,907	1,907	1,907	1,907	1,907	1,907

AOR (Adjusted Odds Ratio) values > 1 indicate higher odds of reporting that the listed motivator did not influence the respondent’s vaccination decision. SE = Standard errors*** *p* < 0.01, ** *p* < 0.05. Motivators analysed:

Available at my place of work during working hours.

Opportunity to ask questions and think about the vaccine before making a decision.

Enough information about the safety of the vaccine.

Information available from official international bodies.

Wanted to visit vulnerable family or friends so had the vaccine so that I would not infect them.

Colleagues from the same profession have had the vaccine.

Leaders/management in my organisation have had the vaccine.

Most of my work colleagues were having the vaccine.

## Discussion

The underlying data on COVID-19 infection in HCW makes VH (at least for COVID-19) seem puzzling. HCWs were at increased risk of hospitalization due to close and long contact with COVID-19 patients and occupational exposure to COVID-19^19,20^. Direct patient-facing HCWs were at higher risk for COVID-19- related outcomes: for example, in one meta-analysis with 119,883 HCWs, 51.7% of HCWs became infected with COVID-19^21^, while another meta-analysis with 230,398 HCWs found that 5% of COVID-19 cases in HCWs had severe complications, and 0.5% of HCWs died^22^. Vaccination against COVID-19 has also been associated with a substantial reduction in absenteeism among healthcare professionals, contributing to improved workforce continuity. A systematic review by Maltezou demonstrated that vaccinated HCWs experienced fewer and shorter episodes of work absence related to COVID-19^23^.

The COVID-19 pandemic had very few benefits but one was the rapid development of safe and effective vaccines, especially when one considers the time it has taken to develop effective vaccinations against other respiratory pathogens^24^. HCW perceptions about vaccines are particularly important as they have a major role in influencing vaccine confidence and hesitancy of the general public; additionally sub-optimal vaccination of HCWs adds further stress to burdened health systems. Many HCWs in this study, despite accepting vaccination, were concerned about the safety of COVID-19 vaccination (and to a lesser extent the efficacy of vaccines offered).

We considered which factors helped reduce VH and encouraged HCWs to be vaccinated against COVID-19 in relation to confidence, complacency and convenience—the Three Cs^8^. Of the Three Cs, “confidence” and “convenience” were most prominent: confidence in the vaccine was represented by high scores for sufficient safety information, especially from independent international organisations and being able to ask questions and were the most important factors overall; ‘convenience’ was demonstrated by the highest score for easy vaccine availability at work, and high proportions citing availability at local clinics, together with the desire to travel (and reduced burden and cost of testing etc. in the unvaccinated).

In practice, therefore, overall vaccination rates would likely be boosted by reducing the ‘friction’ associated with vaccination i.e. promoting a nudge behavioural approach strategy^25,26^which would be reinforced by social cohesion pressures. This is evidenced by the high proportion indicating that their decision to vaccinate was reinforced if colleagues from the same profession and work colleagues, as well as leaders/management in their organisation were vaccinated. Others have shown (supported in our study) that a specific offer of vaccination (e.g. a timed appointment) increases vaccine uptake^27^.

Addressing complacency for COVID-19 vaccination would seem to be of more marginal value for COVID-19. HCWs rightly were concerned that they should be vaccinated to prevent transmission and by listening to international, national and independent professional advice were likely to be vaccinated if they had risk factors associated with disease severity. Also, a high proportion accepted the need for compulsory COVID-19 vaccination for HCWs (including students) who were patient-facing. Indeed some participating countries implemented mandatory COVID-19 vaccination policies for healthcare workers during certain phases of the pandemic. For example, Germany mandated vaccination for HCWs between March and December 2022 and Poland did likewise^28,29^.

However, a smaller proportion had received influenza vaccination in the years before the pandemic and were not necessarily planning to be vaccinated in the year of the study. This was despite HCWs at the centers in Germany, Lithuania, Poland, Portugal and Brazil being offered free influenza vaccination. This together with a much small proportion willing to accept mandatory vaccination suggests that addressing complacency would be of greater importance for vaccination of Influenza and other serious respiratory viral diseases. Future research should explore factors affecting mandatory vaccine acceptance, and differences across cultures, nations, and HCW categories.

Although cost was not an issue here as all centres provided COVID-19 and influenza vaccines for free, previous general population studies have demonstrated that even modest co-payments deter individuals from being vaccinated especially in low-income groups (and many HCWs are in such groups)^30,31^ So, cost especially in LMIC, or in marginalised groups within high income countries (HIC) are likely to be relevant and this will include many sub-groups of HCWs who are on low incomes.

The impact of social media was equivocal. Studies investigating associations between social media utilization and vaccine intentions observed both positive and negative relationships^32^. In our study, for HCWs overall, social media was of importance in persuading many to be vaccinated together with advice from scientists and doctors. Celebrity, religious leader and politician endorsement had minimal effect on promoting vaccination with more limited influence from family members. A study on COVID-19 acceptance and vaccine hesitancy in low and middle-income countries also found that celebrities were seldom regarded as a highly trustworthy source for COVID-19 advice^33^. Another recent study demonstrated that a lack of trust in public sector officials had significantly contributed to low COVID-19 vaccination rates among individuals classified as “high risk“^34^.

Parental opinion or influence is more marginal for HCWs as a group. Arguably this would probably be the greatest distinction with general vaccine promotion campaigns currently which are targeted mainly at children through their parents who make the decision on their child’s behalf.

Based on Hofstede’s cultural dimensions^35^, which measure aspects like individualism, power distance, and uncertainty avoidance, Brazil, with typically higher scores in power distance and uncertainty avoidance, is more inclined to support compulsory vaccination for both COVID-19 and influenza. This reflects a greater acceptance of centralized authority and collective action for public health. Acceptance of vaccination in Brazil may also be explained by the long history of large social programmes /public health drives incentivizing vaccination uptake in lower socioeconomic groups and a marker of human rights and progression^36^. Nevertheless, even here, COVID-19 vaccination revealed real disparities indicating that practical outcomes can vary within a country even when the same policy is agreed e.g. Brazilian municipalities with lower education levels, a higher proportion of Black population, higher Gini index, and worse health service indicators had a greater likelihood of having lower vaccination coverage during the vaccination campaign in 2021–2022^36^.

Germany, known for its lower power distance and higher individualism, leans against compulsory vaccination. This suggests a preference for personal freedom and a pragmatic approach to change and progress, where the population critically assesses the implications of societal and technological shifts^35^. Countries where “Individualism” is less valued (a so-called “we” country), vaccination campaigns seem to be more successful when focusing on shared values (e.g. Spain). If the country value is high (“I” country) the success of a vaccination campaign requires communication focused more on individual benefits (e.g. Italy, France)^35^.

Likewise, countries where “uncertainty avoidance” is high are usually associated with better vaccination campaign results. In countries where there is a high “indulgence” value, vaccination campaigns, especially if the vaccine is communicated as an obligation/restriction (e.g. Sweden, UK) tend to do poorly^35^. Our study highlights the importance of tailoring vaccine promotion strategies to address the diverse concerns of different HCW subgroups, including variations by (sub)occupation, education, and geographical location. Enhancing vaccine acceptance will require targeted approaches that consider these differences and intersect with professional groups, to advance critical public health efforts.

Our study had several strengths. It was a multicenter study, with a non-Anglophone focus using a questionnaire with free text options, careful piloting and designed to ensure participants had full anonymity. Questionnaires used validated instruments and were translated into local languages with further verification of translation. Questions were framed to prioritise understanding of the reasons why participants had accepted (or refused vaccination) and what would encourage vaccination.

It also has limitations. One limitation was the relatively small proportion of nurses participating overall for reasons that remained unclear. We speculate that nursing staff may have been more concerned than medical staff, for example, as to how vaccine refusal would be considered by employers and the possibility of disciplinary action. Whilst promotion of anonymity was essential, center ethics committees were perhaps overly worried about the potential for deductive identification of participants, limiting the number of demographic factors collected.

Free text comments (see Supplementary Information) supported many of the more quantitative conclusions. For example, responses to the question what “other factors helped me in getting the covid vaccine”: many respondents spoke of their duty not to spread infection to patients or family but many indicated that they had been forced or coerced by the state or their employer with the threat of loss of employment. One respondent noted the ideal scenario where their direct supervisor was a doctor who patiently answered all their questions and then left it to their own decision. Clearly a one-to-one or similar approach is very effective but also time-consuming, especially in an emergency situation. There is a need to develop nudge behavioural, social cohesion and similar strategies which can be delivered to larger numbers of HCWs to encourage them to be vaccinated. Arguably there is more time to do this on a regular and continuous basis within HCW training programmes so that a pervading pro-vaccine culture exists across health institutions.

## Supplementary Information

Below is the link to the electronic supplementary material.

Supplementary Material 1

## Data Availability

The authors declare that the data supporting the findings of this study are available within the paper and its Supplementary Information file.
