# Supplementary material for: Determinants of vaccine hesitancy among healthcare workers in an international multicenter study within the EuCARE project
Source: Sci Rep. 2025 Aug 28;15:31703. doi: 10.1038/s41598-025-17507-y (PMC12394626; doi:10.1038/s41598-025-17507-y)
Supplement: Supplementary file 1 — Supplementary Material 1 [file 41598_2025_17507_MOESM1_ESM.docx]

**Summary manuscript changes;**

Affiliations: Please correct the following:

Aff2: Raheelah Ahmad: is now: Department of Global, Public & Population Health & Policy, School of Health & Medical Sciences, City St George’s University of London, Northampton Square, London, EC1V 0HB, UK.

Aff10: Caporali: is now: School of Medicine and Hospital das Clínicas, Universidade Federal de Minas Gerais, Belo Horizonte, Minas Gerais, Brazil

Page 2; Introduction: the first line of the third paragraph should conclude with a full stop after the phrase ‘the problem of VH.’”

Page 6: line 13 (below Table 3) should be: “presented in Fig 2; most common reason being the vaccine was not specifically offered to them..”

Page 7: Table 5. Remove first 8 lines (“Disbeliwf1, 2, 3, 4 etc) as this is a repeat of the same text at the end of the table which should remain.

Page 9: remove “The” in Table 6 sentence. Ie should be “Table 6 is colour coded based on the …..”

Page 11: Discussion: sentence should end with reference 36 not superscript text as current “…..indicators had a greater likelihood of having lower vaccination coverage during the vaccination campaign in 2021-22 ^36^
